# Evaluation of noise pollution impact on health in Dhaka city, Bangladesh

**DOI:** 10.3389/fpubh.2024.1477684

**Published:** 2024-11-15

**Authors:** Masrur Abdul Quader, Md Mostafizur Rahman, Musabber Ali Chisty, Khawla Saeed Al Hattawi, Edris Alam, Md Kamrul Islam

**Affiliations:** ^1^Department of Disaster and Human Security Management, Faculty of Arts and Social Sciences, Bangladesh University of Professionals, Dhaka, Bangladesh; ^2^Department of Disaster Management and Resilience, Faculty of Arts and Social Sciences, Bangladesh University of Professionals, Dhaka, Bangladesh; ^3^Institute of Disaster Management and Vulnerability Studies, University of Dhaka, Dhaka, Bangladesh; ^4^Natural Hazards Center, Institute of Behavioral Science, University of Colorado Boulder, Boulder, CO, United States; ^5^Department of Sociology, University of Colorado Boulder, Boulder, CO, United States; ^6^Faculty of Resilience, Rabdan Academy, Abu Dhabi, United Arab Emirates; ^7^Department of Civil and Environmental Engineering, College of Engineering, King Faisal University, Al-Ahsa, Saudi Arabia

**Keywords:** noise exposure, equivalent continuous sound pressure level (LAeq), noise perception, Dhaka, self-reported health status

## Abstract

**Objectives:**

The purpose of this research was to look at the interrelation between adult health issues in Dhaka and noise pollution.

**Methods:**

The methodology involved a cross-sectional survey conducted in five different land use categories, with a sample size of 1,016 individuals. A validated questionnaire that focused on sources of perceived noise pollution and health issues related to noise was used to gather subjective data for the study. Objective noise pollution was evaluated using equivalent continuous sound pressure level (LA_eq_).

**Results:**

Findings revealed noise generated from road traffic are the predominant source of noise pollution, with Thursday evenings during the end of office hours being the noisiest period in Dhaka. All areas in Dhaka exceeded permissible noise levels, posing significant health risks to residents and workers. The study identifies critical gaps in existing noise regulation policies and enforcement.

**Conclusion:**

Overall, this study underscores the urgent need for comprehensive noise pollution mitigation strategies, including innovative technologies, real-time monitoring systems, and public awareness campaigns. Further studies in diverse urban contexts are recommended to enhance the understanding of noise pollution’s long-term impacts on vulnerable populations.

## 1 Introduction

### 1.1 Noise pollution and its sources

Noise pollution has become a significant environmental concern in many big cities globally (1–4). In Bangladesh, noise pollution is a pressing issue, with levels exceeding human-tolerable thresholds, particularly in large cities like Dhaka (5–7). Road traffic, construction activities, and community events are identified as primary sources of noise pollution in Dhaka. Significantly, all assessed areas consistently exceed acceptable noise levels, with severe health impacts, particularly in mixed-use and industrial areas, as evidenced by a regression analysis linking higher noise exposure to worse health outcomes. Despite regulations from the Department of Environment in Bangladesh, enforcement and implementation remain weak, contributing to excessive noise exposure (7). In 2019, 55% of premature deaths in Bangladesh were caused by various forms of pollution, costing the nation 8.32% of its GDP (8). It is necessary to identify vulnerable areas and individuals both subjectively and objectively. This study investigates the relationship between adult Bangladeshi people’s self-reported health status and observed noise pollution in Dhaka city. The primary hypothesis is that noise pollution is significantly associated with adverse health outcomes among the adult population. When evaluating self-reported health status, we took into account a number of noise-related health concerns that have been identified in other investigations (9–11). There have been several prior research on noise pollution in Bangladesh (5, 6, 12, 13). This is the first study to explore health issues identified through self-reported health methods among adults in Bangladesh and actual observed noise pollution levels. The exploration of this study could be beneficial in creating strategies to reduce noise pollution at local and national levels.

Globally, noise pollution is an escalating concern, with transportation—especially road traffic, air travel, and shipping—being a significant contributor. Shipping alone, accounting for 80% of global trade, generates persistent underwater noise that disrupts marine ecosystems (14). Urban areas are especially affected, with cities like New York exposing 90% of mass transit users to harmful noise levels that contribute to serious health issues like hearing loss and cardiovascular disease (15). Noise pollution not only impacts human health but also disrupts wildlife, affecting species’ communication, navigation, and survival (16).

In response, advanced regulatory frameworks like the European Union’s Environmental Noise Directive (END) have been established. These frameworks mandate strategic noise mapping (SNM) to help identify and control noise pollution in different regions (17). Complementary measures, such as urban green spaces and noise barriers, are being integrated into urban planning to mitigate noise levels.

Locally, cities in developing nations like Dhaka face significant challenges with noise pollution due to rapid urbanization and lax enforcement of existing laws (18). Similar challenges are observed in Mumbai, where high population density contributes to noise issues. However, Mumbai has implemented urban planning strategies using green spaces to reduce noise (19). Given Dhaka’s lack of green infrastructure, such measures could be highly beneficial. In contrast, Lagos faces aggravated noise pollution issues due to insufficient resources and ineffective regulation enforcement amidst rapid growth (20). Both Dhaka and Lagos experience difficulties in enforcing noise control measures, while Mumbai’s example highlights the potential of integrating green infrastructure as part of the solution (18, 21).

Incorporating green spaces, boosting public awareness, and enforcing regulations more rigorously could significantly improve the local noise pollution situation in cities like Dhaka and Lagos (19–21). Global innovations and regulatory practices can serve as models for local adaptations to manage noise pollution effectively.

### 1.2 Impact of noise pollution on public health

Noise pollution has been linked to various health risks such as cardiovascular problems, cognitive impairments, and mental health issues (10, 22–24). Elongated loud noise pollution (over 70 dB(A)) can be dangerous to one’s mental as well as physical health over time (25, 26). Environmental stressors like deafness, sleeplessness, high blood pressure, heart disease, headaches, stress, poor focus, decreased productivity, exhaustion, irritability, indigestion, heartburn, and ulcers have been linked to it (11). Noise pollution is considered the second-highest burden of health effects after air pollution (25, 27).

People’s perceptions of noise pollution impact their quality of life, health, and mobility decisions (9, 28). Self-reported health is a crucial measure of an individual’s psychological, physical, and social well-being, which is not easily quantified by a single health issue metric. Poor self-reported health is strongly correlated with early life loss (13, 28). However, a variety of health status brought on by different environmental, economic and social factors may be included in self-reported health surveys. For example, questionnaires from most self-reported health just have one simple question indicating on, how would one evaluate their general health. The answer in them is scored in three to five scores parameters (fair, medium or poor health condition) (29, 30). Health status may be influenced by socioeconomic level and environmental issues like waste, noise, and air pollution (28). Self-reported health is vital for understanding the holistic impact of environmental factors on individuals’ well-being, as it captures personal perceptions of physical, mental, and social health, offering insights beyond clinical measures and contributing to quality-of-life assessments (31). It also considers contextual nuances such as social environment, workplace conditions, and cultural differences, enhancing the understanding of how environmental stressors like noise pollution affect health over time (13, 32). Studies show the importance of both subjective and objective measures in understanding the health effects of noise pollution (9, 13, 28, 33). Former provides quantifiable data on exposure levels, while latter capture individual perceptions and real-world effects on well-being (34, 35).

Noise pollution is considered the second-highest burden of health effects after air pollution (25, 27). Noise pollution in Bangladesh poses a substantial threat to public health, with studies showing that almost 12% of the population has suffered hearing loss due to excessive noise exposure (7).

### 1.3 Research gaps and study objective

#### 1.3.1 Research gaps

Bangladesh has conducted numerous studies on noise pollution in the past (5, 12, 13), but none have examined the relationship between self-reported health effects and measured noise pollution levels. Unfortunately, since current studies primarily apply accurate measurements of noise levels, there is no comprehensive approach linking these measures to the public’s perception of health. This study meets this need by integrating self-rated health with observed, quantifiable noise data, enabling an evaluation of the overall effects of noise pollution on Dhaka city residents. In order to determine the effect of noise pollution on public health in Dhaka city, this study compares collected data on noise pollution with self-reported health.

#### 1.3.2 Study objectives

The major goals are to analyses variations in noise pollution levels according to land-use and provide policy makers with specific recommendations on actions that should be taken to reduce noise pollution in the interest of health.

- To compare the perceived health effects of noise pollution in different parts of Dhaka city with self-reported health effects.
- To evaluate how noise pollution varies according to Dhaka’s various land uses.
- To provide recommendation guidelines to help all policy makers to minimize the adverse health effects that arise from noise pollution.

The findings of this study can aid in the development of the long-term goals of the nationally and locally developed plan to address noise pollution. To better understand the effects of noise pollution on public health, we have taken into account in this work the correlation between real noise and self-reported health.

## 2 Methods

**Figure 1:**
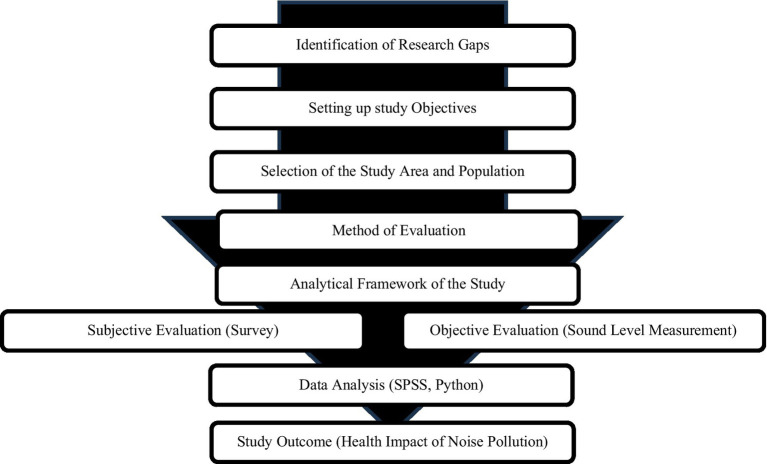
Methodological flowchart of the study (Dhaka, Bangladesh, 2023).

**Figure 2:**
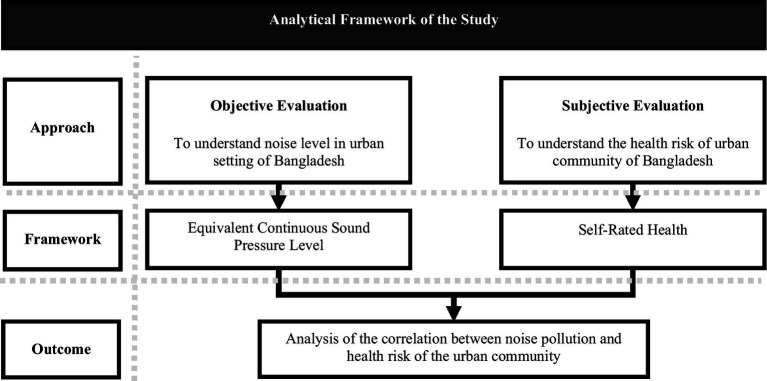
Analytical framework of the study (Dhaka, Bangladesh, 2023).

The flowchart (Figures 1, 2) outlines the methodological framework of the study, beginning with the identification of the research gap on the health impacts of noise pollution in Dhaka. It proceeds through the study design, which includes defining objectives, selecting study areas based on land use classifications, and employing both subjective and objective data collection methods. Subjective observations involved participant surveys on health perceptions related to noise exposure, while objective observations included direct noise level measurements across selected areas. The analysis integrates both data sources to assess health outcomes, providing a holistic evaluation of noise pollution’s effects.

This cross-sectional study surveyed adult residents of Dhaka city to understand their perceptions of health issues related to noise. The research adhered to ethical concerns and was approved by the Research Ethics Review Committee of the Institute of Disaster Management and Vulnerability Studies at the University of Dhaka (Ref. ERC-02/020221). Participants provided consent, and the questionnaire stated that the information would only be used for research, without any incentives for participation.

### 2.1 Study areas

The study focused on Dhaka city and selected five regions based on land use classifications in the Road Transport Act, 2018, and the Environment Conservation Rules, 1997 (Table 1) for further analysis, as presented in Table 2.

**Table 1:** Acceptable noise limit according to land use pattern.

Land use pattern	Daytime dB(A)	Night-time dB(A)
Silent zone (Sensible area)	45	35
Residential area	50	40
Mixed-use area	60	50
Commercial area	70	60
Industrial area	75	70

Source: ECR (49); RTA (7).

**Table 2:** Study area according to land use category (Dhaka, Bangladesh, 2023).

SL no	Category of area	Selected area
A	Silent zone (Sensible area)	Dhaka Medical College Area (DMC)
B	Residential area	Azimpur Residential Area
C	Mixed-use area	Shiddeshori
D	Commercial area	Motijheel
E	Industrial area	Tejgaon

### 2.2 Data collection and sampling

The study conducted a one-to-one survey between February and mid-March 2023, focusing on noise levels in five land use categories. The survey was conducted among individuals aged 18 and above who had lived or visited these locations for at least 5 years. Morgan’s Table indicated that for this perception-based investigation, a minimum of 384 participants in the sample (confidence interval: 95%) was required (36). A non-probability sampling strategy was used and covered 1,016 respondents for precision. Observed noise levels were collected simultaneously across the five land use categories, 7 days a week, avoiding any special occasions to avoid distorted noise readings. A pilot on noise level measurement was conducted to identify suitable locations for each category.

### 2.3 Subjective data collection

The questionnaire was developed based on extensive literature review [e.g., (11, 13)] and existing surveys on noise pollution perception and health effects. Questions were designed to assess participants’ social status, demographic data, perceived sources of noise pollution, and related health issues. A pilot survey among 50 university students was conducted to validated a draft questionnaire for a study on noise pollution (13). Minor revisions were made based on their responses to enhance clarity and ensure the questions were understandable to the general population. The pilot testing helped refine the wording of certain questions to enhance clarity, ensuring they were easily understandable by the general population. Experts in public health and noise pollution examined the questionnaire to make sure the items accurately reflected the concepts being measured. However, formal reliability testing, such as Cronbach’s alpha to assess internal consistency across items, was not conducted in this study. The lack of reliability testing may limit the study’s ability to confidently assess the internal consistency of the instrument. Future research should incorporate formal reliability and test–retest reliability methods to ensure consistency and stability of responses over time.

The questionnaire, available in both Bangla and English, covered social status, demographic data, sources of perceived noise pollution, and health issues related to noise (11), with responses scored as 1 (positive), 0.5 (neutral), or 0 (negative) (13). Participants self-reported thirteen noise-related health issues and were asked about the types and main noise pollution sources in their area. The questionnaire was created and uploaded in Google Forms for secure collection, protection, and cloud storage. Measures to reduce bias included standardizing the survey process: all interviewers received training to avoid introducing interviewer bias by maintaining neutrality during the interview process. In order to ensure that participants had long-term exposure to and memory of noise difficulties, recall bias was reduced by only included those who had resided in or visited the study regions regularly for at least 5 years. By incorporating enquiries about participants’ medical histories, confounding factors—like pre-existing health conditions—were addressed and made controllable throughout the data analysis stage. In order to separate the impact of noise pollution on health outcomes during data analysis, confounders including pre-existing medical disorders were taken into account in the linear regression models.

### 2.4 Objective data collection

The study used a UNI-T UT351 Digital Sound Level Meter to gather objective data on noise pollution levels in five selected locations within the study areas (37). The study included five selected locations within the study areas, representing the different land use categories. A pilot study was conducted to identify the most suitable locations for noise measurement within each category to ensure accurate representation of the noise levels. Noise levels were recorded 7 days a week, and the data was documented manually for analysis.

### 2.5 Analysis framework for objective evaluation and health status

The study evaluated noise pollution using equivalent continuous sound pressure level (LA_eq_) with alignment to several studies (38–42) and incorporated subjective evaluation through a self-rated health model to establish a correlation between noise exposure and urban health status.

#### 2.5.1 The equivalent continuous sound pressure level (LAeq)

The study assessed the LA_eq,_ LA_90_ (noise level exceeded for 90% of the testing duration) and LA_10_ (noise level surpassed for 10% of the measuring time) levels of sound energy and level, as they could change significantly depending on time and place (31). The LA_eq_ is a uniform, stable sound with the same energy level as non-uniform sounds.

The following formula has been utilized to determine LA_eq_ (43).

$$LA_{\mathrm{eq}}=10\log_{10}\overset{n}{\underset{i=1}{\sum }}\left(10^{\frac{Li}{10}}\right).\;ti$$

Where, LA_eq_ = Equivalent Continuous Sound Pressure Level, Li = Noise Weighted in dB(A), ti = Time period over which (LA_eq_).

#### 2.5.2 Self-rated health (SRH) model

Self-rated health (SRH) is a subjective assessment of an individual’s health status, encompassing biological, mental, social, and functional dimensions. Developed in 1958 by Suchmann, Phillips, and Streib, SRH is a pragmatic tool for assessing overall health status and providing a concise snapshot of an individual’s health perception (44, 45). It is closely tied to prosperity and quality of life and has proven to be a strong predictor of mortality. SRH is useful in clinical practice as it captures vital health information that may be difficult to obtain through lengthy surveys or examinations (46, 47). A study in Finland found that SRH could predict short-term mortality risk (less than 10 years) similarly to objective health status (44). Another study estimated life expectancy adjusted by self-rated health status, emphasizing SRH’s role in understanding overall health and longevity (48).

### 2.6 Noise acceptance limit of Bangladesh

The Environmental Conservation Rule of 1997, the Noise Pollution (Control) Rules of 2006, and the Road Transport Act of 2018 all classify the areas of noise level according to the land use category. These areas are divided into five classes: the silent zone (sensible area), industrial area, residential area, commercial area and mixed-use area. Together with the various time intervals of the day, the dB(A) amount in these regions is also indicated (Table 1). Data were gathered for the study 7 days a week at four different times of the day: office hours, which are from 9 am to 11 am, school playtime, which is from 12 pm to 4 pm, office hours, which are from 5 pm to 8 pm and midnight, which is from 12 am to 1 am (49–51).

### 2.7 Data analysis

The study used IBM SPSS Version 26 and Python (version 2.7; Beaverton, OR 97008, USA) for statistical analysis and data management (52, 53). Descriptive statistics were computed, and the overall score for health issues associated with noise pollution was estimated. The total health concerns associated with noise may indicate a person’s health issues caused by noise pollution. A linear regression analysis examined factors influencing self-reported overall noise-related health issues, using the 95% confidence interval (CI) in all statistical analyses. Before running the regression models, key assumptions for hypothesis testing were checked. The normality of residuals was tested using the Shapiro–Wilk test, and independence of errors was assessed with the Durbin-Watson statistic. Homoscedasticity was evaluated through residual vs. fitted values plots to check for constant variance of errors. These diagnostic tests ensured that the model met the assumptions for valid inference.

For objective noise level measurements, the Equivalent Continuous Sound Level (LA_eq_) was calculated. The noise level that exceeded 10% of the measurement duration (LA_10_) and 90% of the measurement duration (LA_90_) was also determined to account for variations in noise levels across different times and locations.

## 3 Results

**Figure 3:**
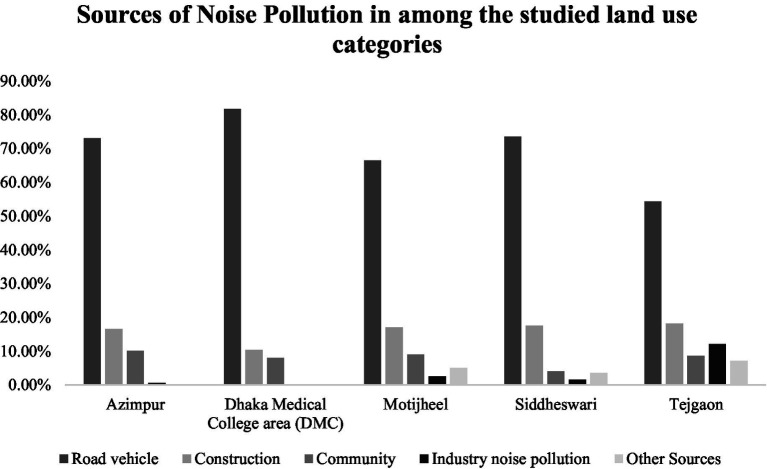
Sources of noise pollution among the studied land use categories (Dhaka, Bangladesh, 2023).

Dhaka’s urban landscape is characterized by significant noise pollution, with traffic noise being the primary source. Construction activities and community events also contribute to the noise levels, ranking third and fourth, respectively. Industrial zones in Dhaka have a unique soundscape, with machinery hums and clanks. The distant roars of airplanes and trains also enrich the urban noise, making it a significant concern in the city. The vibrancy of community events, such as markets and festivals, also contributes to the ambient noise environment. Overall, Dhaka’s urban noise pollution is a significant issue (Figure 3).

Table 3 shows noise levels for five land use patterns. The highest daytime noise (LA_90_) was 111.6 dB(A) in Azimpur (residential area), while the highest nighttime noise (LA_90_) was 107.3 dB(A) in Motijheel (commercial area). LA_eq_ exceeded acceptable levels in all areas (49, 51), with DMC (Sensible Area) having the highest pollution, exceeding thresholds by 33 dB(A) during the day and 38 dB(A) at night. Azimpur exceeded levels by 28 dB(A) during the day and 27 dB(A) at night (Table 4).

**Table 3:** Comparability of noise levels among the studied land use patterns (Dhaka, Bangladesh, 2023).

Land use pattern	Period	Noise level description [dB(A)]		
		LA_10_	LA_90_	LA_eq_
Tejgaon (Industrial Area)	Day	64.4	107.9	84
	Night	64.9	103.4	80
Motijheel (Commercial Area)	Day	60.9	109.4	83
	Night	53.8	107.3	81
Shiddeshori (Mixed use Area)	Day	61.7	106.2	84
	Night	51.5	101.2	71
Azimpur (Residential Area)	Day	50.2	111.6	78
	Night	42.2	99.8	67
DMC (Sensible Area)	Day	51.4	108.9	78
	Night	51.2	102.3	73

**Table 4:** Comparison of noise levels among the different time categories in the five-category area studied (Dhaka, Bangladesh, 2023).

Area	Industrial area	Commercial area	Mixed-use area	Residential area	Sensible area
Office Hours (9:00 AM-11:00 AM)	65.5	61.6	63	51.2	53.2
	107.9	106.4	106.2	111.6	104.4
	84	83	84	78	76
School Recess (12:00 am-4:00 pm)	64.6	62.2	62.1	50.2	51.4
	104.8	108.1	105.3	107.7	108.9
	83	82	84	77	78
End of Office Hour (5:00 pm-8:00 pm)	64.4	60.9	61.7	50.6	60.2
	107.6	109.4	106.2	107.7	105.5
	84	83	84	78	80
Mid Night (12:00 AM-1:00 AM)	64.9	53.8	51.5	42.2	51.2
	103.4	107.3	101.2	99.8	102.3
	80	81	71	67	73

Industrial Area = Tejgaon.

Commercial Area = Motijheel.

Mixed-use Area = Shiddeshori.

Residential Area = Azimpur.

Sensible Area = Dhaka Medical College Area.

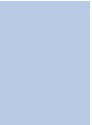
￼ LA10 = Noise level exceeded 10% of the measurement period.

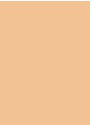
￼ LA90 = Noise level exceeded 90% of the measurement period.

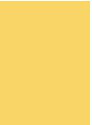
￼ LAeq = Equivalent Continuous Sound Level.

The noise levels in five urban areas of Dhaka, including Tejgaon, Motijheel, Shiddeshori, Azimpur, and DMC, significantly exceed regulatory thresholds. Measurements were taken at midnight, end of office hours, school break, and office hours. These levels are compared with legal thresholds from the Road Transport Act of 2018 and Environment Conservation Rules of 1997.

During office hours, LA_eq_ in Tejgaon is 84 dB(A), 9 dB(A) over the 75 dB(A) limit. Motijheel is 83 dB(A), 13 dB(A) over the 70 dB(A) limit. Shiddeshori records 84 dB(A), 24 dB(A) over the 60 dB(A) limit. Azimpur is 78 dB(A), 28 dB(A) over the 50 dB(A) limit. DMC is 76 dB(A), 31 dB(A) over the 45 dB(A) limit.

During school recess, Tejgaon is 83 dB(A), 8 dB(A) over the limit. Motijheel is 82 dB(A), 12 dB(A) over. Shiddeshori records 84 dB(A), 24 dB(A) over the 60 dB(A) limit. Azimpur is 77 dB(A), 27 dB(A) over. DMC is 78 dB(A), 33 dB(A) over the 35 dB(A) limit.

The noise level measurements for five categorical areas, including office hours, school recess, end of office hours, and midnight, are presented in Tables 5–9, categorized by the Road Transport Act of 2018 and the Environment Conservation Rules of 1997. The measurements are presented in dB(A) and are based on different days and time categories.

**Table 5:** Comparison of noise levels among the different time categories in the industrial area (Tejgaon) (Dhaka, Bangladesh, 2023).

Day	Friday	Saturday	Sunday	Monday	Tuesday	Wednesday	Thursday
Office Hours (9:00 AM-11:00 AM)	67.1	66.6	66.3	67.8	67.3	67.1	65.5
	101.7	100.8	103.7	106.3	107.9	107	102
	82	81	83	86	86	85	83
School Recess (12:00 am-4:00 pm)	65.4	64.6	66.8	67.4	67.2	66.8	67.1
	101.6	103.7	103.1	104.5	103.7	104.6	104.8
	80	84	83	84	83	84	83
End of Office Hour (5:00 pm-8:00 pm)	65.6	64.4	65.4	65.6	65.2	65.3	67
	102.2	104.2	106.3	104.8	107.6	103.6	105.9
	80	84	84	84	85	85	86
Mid Night (12:00 AM-1:00 AM)	68	67.4	68.6	69.6	69.8	67.7	64.9
	101.7	99.8	86.6	103.4	101.5	101.1	97.3
	81	79	77	83	82	80	80

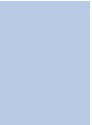
￼ LA10 = Noise level exceeded 10% of the measurement period.

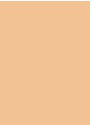
￼ LA90 = Noise level exceeded 90% of the measurement period.

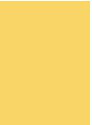
￼ LAeq = Equivalent Continuous Sound Level.

**Table 6:** Comparison of noise levels among the different time categories in the commercial area (Motijheel) (Dhaka, Bangladesh, 2023).

Day	Friday	Saturday	Sunday	Monday	Tuesday	Wednesday	Thursday
Office Hours (9:00 AM–11:00 AM)	61.6	62.4	63.5	66.2	65.4	65.5	64.9
	102.6	102.9	103.4	102.8	106.1	104.5	106.4
	80	81	83	84	85	85	85
School Recess (12:00 am–4:00 pm)	62.4	62.2	62.5	63.5	64.7	65.3	63.2
	101.2	102.5	102.1	108.1	105	104.9	104.5
	79	79	81	82	84	84	84
End of Office Hour (5:00 pm-8:00 pm)	61.9	64.5	66.2	63.6	64.7	64.5	60.9
	102.6	102.6	105.3	105.1	106	105.5	109.4
	80	81	85	84	83	84	84
Mid Night (12:00 AM-1:00 AM)	54.2	54.6	60.1	54.6	53.8	62.6	61.6
	104	103.5	103.7	107.3	103.6	104.2	103.1
	80	80	81	82	82	83	80

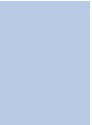
￼ LA10 = Noise level exceeded 10% of the measurement period.

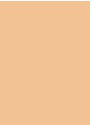
￼ LA90 = Noise level exceeded 90% of the measurement period.

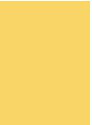
￼ LAeq = Equivalent Continuous Sound Level.

**Table 7:** Comparison of noise levels among the different time categories in mixed-use area (Shiddeshori) (Dhaka, Bangladesh, 2023).

Day	Friday	Saturday	Sunday	Monday	Tuesday	Wednesday	Thursday
Office Hours (9:00 AM-11:00 AM)	63.5	65.5	63	63.2	63.7	65.4	66.4
	103.9	102.5	104.1	103.4	103.9	103.1	106.2
	81	83	84	84	84	84	86
School Recess (12:00 am-4:00 pm)	62.1	66.2	62.8	63.2	62.9	65.9	66
	105	104.2	104.1	103.4	104.2	105.3	104.1
	82	85	84	84	85	86	86
End of Office Hour (5:00 pm-8:00 pm)	64.8	64.3	61.7	62.9	64.1	63.2	65.4
	102	104.8	105.1	104.3	105.1	105.8	106.2
	80	85	84	84	86	85	87
Mid Night (12:00 AM-1:00 AM)	51.5	59.7	58.4	58.4	55.9	53.7	55.4
	101.2	89.3	81.7	84.8	83.8	89.4	86.8
	76	73	70	71	69	70	70

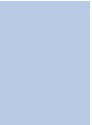
￼ LA10 = Noise level exceeded 10% of the measurement period.

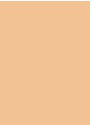
￼ LA90 = Noise level exceeded 90% of the measurement period.

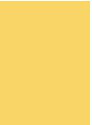
￼ LAeq = Equivalent Continuous Sound Level.

**Table 8:** Comparison of noise levels among different time categories in the residential area (Azimpur) (Dhaka, Bangladesh, 2023).

Day	Friday	Saturday	Sunday	Monday	Tuesday	Wednesday	Thursday
Office Hours (9:00 AM-11:00 AM)	51.7	51.2	55.2	51.2	51.5	51.2	51.2
	111.6	102.3	108.1	102.8	107.2	103.1	105.9
	82	77	80	77	77	77	78
School Recess (12:00 am-4:00 pm)	50.3	51.2	50.7	51.7	51.8	50.2	50.5
	103.2	107.7	106.5	103.9	107.1	102.8	105.1
	76	78	78	79	77	76	77
End of Office Hour (5:00 pm-8:00 pm)	51.6	51.5	50.8	51.7	51.8	51.3	50.6
	106.5	105.1	107.7	104.8	105.7	105.1	107.1
	78	77	77	78	78	78	78
Mid Night (12:00 AM-1:00 AM)	43.7	42.2	45.9	43.1	45.4	44.3	45.2
	99.8	90.7	91.5	86.6	88.1	87.5	88.1
	69	67	67	65	68	65	67

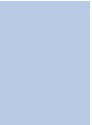
￼ LA10 = Noise level exceeded 10% of the measurement period.

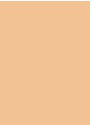
￼ LA90 = Noise level exceeded 90% of the measurement period.

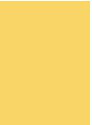
￼ LAeq = Equivalent Continuous Sound Level.

**Table 9:** Comparison of noise levels among the different time categories in Sensible Area (Dhaka Medical College) (Dhaka, Bangladesh, 2023).

Day	Friday	Saturday	Sunday	Monday	Tuesday	Wednesday	Thursday
Office Hours (9:00 AM-11:00 AM)	53.2	60.4	60.4	60.6	62.1	61.6	61
	86.4	88.2	88.9	100.4	102.9	89.4	104.4
	71	73	73	76	79	75	81
School Recess (12:00 am-4:00 pm)	51.4	60.4	60.5	60	61.6	60.8	60.2
	102.5	99.6	104.2	101	104.2	108.9	108.6
	76	76	79	77	78	79	80
End of Office Hour (5:00 pm-8:00 pm)	60.2	63	61.6	61.3	65.2	60.7	62.7
	102.9	104	104.2	105.5	102.3	102.8	105.4
	76	79	80	80	81	81	82
Mid Night (12:00 AM-1:00 AM)	52.3	51.2	56.2	52.7	54.6	52.7	61.2
	84	87.8	89.2	83.7	101.7	101.2	102.3
	68	71	73	68	77	73	80

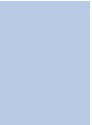
￼ LA10 = Noise level exceeded 10% of the measurement period.

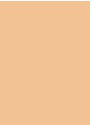
￼ LA90 = Noise level exceeded 90% of the measurement period.

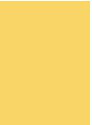
￼ LAeq = Equivalent Continuous Sound Level.

The study reveals that Monday and Tuesday are the noisiest days in Tejgaon, particularly during office hours, with a noise level of 86 dB(A). On Thursday, the same level is observed during the office closing hour. Despite slight reductions during weekends, noise levels consistently exceed the acceptable industrial threshold of 75 dB(A). Office closing hours on Tuesday and Wednesday are the second noisiest time of the week. On Mondays, noise levels remain high, with an LA_eq_ of 83 dB(A) during school recess, 84 dB(A) at the end of office hours, and a slight drop to 83 dB(A) at night. On Tuesdays, the LAeq is 83 dB(A) during school recess, 85 dB(A) at the end of office hours, and 82 dB(A) at midnight. Fridays see a further reduction in noise levels, as all offices and schools remain closed on weekends, reducing traffic stress. On Saturdays, a large number of offices and urban areas remain closed, but some schools remain open. Sundays have an LA_eq_ of 83 dB(A) during office hours, 83 dB(A) during school recess, 84 dB(A) at the end of office hours, and a noise level at its lowest around midnight, measuring 77 dB(A).

The study reveals that Tuesdays to Thursdays are the noisiest days in commercial areas, especially during office hours, with a noise level of 85 dB(A), significantly higher than the acceptable 70 dB(A) threshold. On Sundays, the office hour has a noise level of 83 dB(A), but the highest level is 85 dB(A) during office closing hours. Commercial areas face high noise levels during the five working days of the week, with most noise occurring during office and closing hours (Table 6). At midnight, the noise levels drop slightly to 80 dB(A), but remain above the acceptable nighttime threshold of 60 dB(A). Wednesdays have the highest midnight noise level of the week, likely due to ongoing commercial activities or late-night traffic.

The mixed-use area in Shiddeshori consistently exceeds regulatory noise limits, with Thursdays being the most polluted day. The LA_eq_ values are highest during office hours and school recess, with Thursdays reaching 86 dB(A), significantly higher than the acceptable threshold of 60 dB(A) during the daytime (Table 7). These high levels are consistent throughout the day and reflect the mixed-use nature of the area, combining residential, commercial, and traffic noise. School recess hours were the most noise-polluted area throughout the week, with the lowest being 84 dB(A) on Sunday and Monday and the highest being 86 dB(A) on Thursday. At the end of office hours, LAeq values remain high, with Thursday showing the highest level at 87 dB(A), followed by Tuesday (86 dB(A)) and Wednesday and Saturday both at 85 dB(A). Midnight noise levels are relatively lower but still exceed the acceptable nighttime threshold of 50 dB(A). The highest LAeq at midnight is recorded on Friday at 76 dB(A), followed by Saturday at 73 dB(A). Other days show slightly lower levels, with Sunday, Monday, Tuesday, and Wednesday ranging between 69 dB(A) and 71 dB(A).

The residential area in Azimpur consistently exceeds regulatory noise limits, with Fridays being the noisiest day of the week (Table 8). Fridays and Sundays are the noisiest days, particularly during office hours, with LA_eq_ values reaching 82 dB(A) and 80 dB(A), respectively, exceeding the acceptable residential area threshold of 50 dB(A) during the daytime. Weekends are also the noisiest. During office hours, LA_eq_ values remain elevated, with the highest levels recorded on Friday (82 dB(A)) and Sunday (80 dB(A)). School recess shows a similar pattern, with Friday recording an LAeq of 76 dB(A) and Monday reaching 79 dB(A). The noise levels on Saturday, Tuesday, and Thursday are consistent at 78 dB(A), while Wednesday records slightly lower levels at 76 dB(A). At the end of office hours, LAeq values remain consistently high, with 78 dB(A) recorded on most days. Midnight noise levels are relatively lower but still exceed the acceptable nighttime threshold of 40 dB(A).

The Dhaka Medical College’s Sensible Area experiences consistently high levels of noise pollution, surpassing regulatory limits on all days and time categories (Table 9). Thursday is the most noise-polluted day, particularly during office hours, with the highest LAeq values. This indicates significant environmental noise issues in this sensitive area. The LAeq values consistently exceed the acceptable threshold of 50 dB(A) during the daytime. School recess hours and office closing hours also show high levels of noise pollution, with the LAeq peaking at 80 dB(A) on Thursday. Other days, such as Sunday and Wednesday, also show high levels. At the end of office hours, LAeq values remain high, particularly on Thursday and Tuesday and Wednesday. Other days, such as Sunday and Monday, show LAeq values of 80 dB(A) each. Friday records a slightly lower level at 76 dB(A). The consistent noise levels in the evening indicate significant environmental noise challenges in this sensitive area.

The survey revealed a diverse demographic distribution among respondents, with service holders making up 40% of the total. Business owners and students made up 25% each, demonstrating a balanced representation across occupational sectors. The remaining portion consisted of unemployed individuals.

The regression analysis conducted in Table 10 aimed to evaluate the connection between noise pollution and health consequences across different urban areas. The dependent variable, health score, was determined based on the presence (scored as 1) or absence (scored as 0) of 13 WHO-recommended health conditions (11, 13), averaged to obtain an overall health score for each area. The health data were collected through individual surveys among the area’s people. The independent variable was the equivalent continuous noise level (LA_eq_) measured in decibels (dB(A)) for the respective areas.

**Table 10:** Regression analysis- correlation of noise exposure and health problems (Dhaka, Bangladesh, 2023).

			Overall Noise-Related Health Problem		
Features	LA_eq_ (dB(A))	Average Health Score	*R*^2^	B# (95% CI)	*p*-value
Location					
- Motijheel (Commercial area)	82.75	3.87		Reference	
- Azimpur Residential area (Residential area)	77.76	3.07		0.00024 (−0.001; −0.000)**	0.0011
- Dhaka Medical College area (Silent zone as sensible area)	77.62	4.34	0.05	0.00024 (−0.000; 0.001)	0.0537
- Shiddeshori (Mixed Area)	84.23	4.71		0.00022 (0.000; 0.001)***	0.0007
- Tejgaon (Industrial area)	83.55	4.31		0.00023 (−0.000; 0.001)	0.0749

#Beta; *p < 0.05; **p < 0.01; ***p < 0.001.

The commercial area (Motijheel) served as the reference location, with an average LA_eq_ of 82.75 dB(A) and an average health score of 3.87. The residential area (Azimpur) had a lower average LA_eq_ of 77.76 dB(A) and an average health score of 3.07 but was found in second place (*B* = 0.00024, 95% CI: −0.001; −0.000) after the mixed-use area (Shiddheshori) (*B* = 0.00022, 95% CI: 0.000; 0.001) with a LA_eq_ of 84.23 dB(A) and an average health score of 4.71. The sensible area (Dhaka Medical College) reported an average LA_eq_ of 77.62 dB(A) and a health score of 4.34, which has been found to have a marginally significant positive relationship with health score (*B* = 0.00024, 95% CI: −0.000; 0.001). The industrial area (Tejgaon) had an LA_eq_ of 83.55 dB(A) and an average health score of 4.31, which was found to be the least affected due to noise exposure (*B* = 0.00022, 95% CI: −0.000; 0.001).

The regression analysis reveals a significant relationship between noise levels (LA_eq_) and health scores, with areas experiencing higher noise levels generally reporting worse health outcomes. The significance of the regression coefficient and *p*-values indicate that noise pollution substantially influences public health, particularly in mixed-use and industrial areas.

## 4 Discussion

According to our research, Dhaka’s traffic noise is the main cause of noise pollution. According to research by Razzaque et al. (54), traffic noise alone is responsible for a substantial 75% of noise pollution, with loudspeakers, cars, and religious events being the main contributors (54). Dey (55) emphasized that one of the main things aggravating the issue is driver ignorance, inexperience, and chaotic traffic conditions.

According to our findings, Dhaka’s noise levels continuously surpass allowable thresholds; in residential areas, the maximum daytime noise level (LA_90_) is an astounding 111.6 dB(A). It emphasizes how seriously noise pollution affects locals. The Motijheel business district had the highest noise level at night, 107.3 dB(A), suggesting persistent noise disruptions during the most important sleeping hours. These results are supported by secondary sources, which report that Dhaka has an average noise frequency of 119 dB, exceeding international health standards and ranking high among the 61 largest cities in the world regarding noise pollution (56). Additional measurements made during business hours at different city locations confirm the ubiquitous effect of traffic noise on Dhaka’s urban environment (54).

According to our research, Thursday has the highest noise pollution levels in Dhaka, with consistently high noise levels recorded at various locations and times. Thursdays are often associated with increased commercial activities and traffic congestion in Dhaka, as they precede the weekend, leading to heightened noise levels due to people closing weekly deals and preparing for the holiday. Particularly concerning is the time frame from 5:00 pm to 8:00 pm, which corresponds with the conclusion of office hours and exhibits consistently high noise levels. One study found that the ongoing effort to lessen these effects is the creation of noise maps, which visually represent the distribution of noise across particular times and locations. For example, during peak hours (09:00–11:00), the noise levels on major roads range from 78.1 dB to 119.7 dB, significantly higher than the permissible limits. Noise densities, which range from 57.4 dB to 89.3 dB, are still significant even during off-peak hours (12:00–15:00) (57).

In comparison, Dhaka’s pollution index of 94.18 far exceeds Mumbai’s 83.50, highlighting more severe noise pollution in Dhaka. Additionally, dissatisfaction with green spaces is higher in Dhaka (77.26) compared to Mumbai (65.78), exacerbating environmental stressors (58). These comparisons underscore the unique urban challenges Dhaka faces.

Our results prove a relationship between health scores and equivalent continuous noise level (LA_eq_), whereby higher LA_eq_ values are typically linked to higher health impact scores. This positive correlation between noise pollution and health effects highlights numerous and extremely problematic effects of high noise levels. Prolonged exposure to high decibel levels has been associated with increased aggression, sleep disturbances, hypertension, stress, and auditory damage (55). Due to their extended exposure to traffic noise, certain demographic groups—drivers and traffic police—are even more vulnerable (59).

Studies have indicated a dose–response relationship between psychological distress, sleep disturbance, and noise annoyance caused by traffic noise (60). Additionally, studies indicate that lower socioeconomic status is associated with higher exposure to traffic noise, leading to increased health risks (61). Similar dynamics have been observed in Southwest Detroit and other places where air and noise pollution are chronically present for the citizens (62). Studies indicate that communities of color and low-income groups experience higher levels of noise pollution compared to wealthier, predominantly white neighborhoods (63). It implies that noise exposure increases the frequency and severity of adverse health effects. Beyond being annoying to listen to, noise pollution has more serious negative health effects. Studies that follow people over time have begun to find links between noise pollution and mental health. For example, one study found a link between mental health outcomes and high levels of urban noise exposure (35). While the connection between noise and cardiovascular problems has been well-documented, our understanding of its effects on mental health is less comprehensive (60). Though the research is still in its early stages, a systematic review and meta-analysis point to a possible connection between noise irritation and worse mental health (64). For instance, a systematic assessment of research from North America demonstrates that certain environmental dangers, such as noise pollution, disproportionately harm communities with lower socioeconomic level (65). Beyond short-term health impacts, mental health symptoms have been linked to the general urban living environment, which is marked by air quality, noise pollution, and other elements. To investigate the connection between urban environmental profiles and psychiatric symptoms, a robustness and reliability analysis employing sparse Canonical Correlation Analysis (sCCA) has been conducted (66).

These findings not only align with previous research but also point to an urgent need for stronger policy interventions. Although Bangladesh has the Noise Pollution (Control) Rules 2006 in place, recent studies suggest significant gaps in enforcement, with urban noise levels consistently exceeding safe limits (67). According to the current policy framework, permissible noise levels vary by zone—silent, residential, and commercial areas—but consistent violations, coupled with inadequate monitoring, weaken the policy’s effectiveness (68).

The Bangladesh Environment Conservation Act of 1995 established the Noise Pollution (Control) Rules of 2006, however their implementation is still noticeably inadequate. The penal provisions, such as fines and a maximum three-year jail sentence, are insufficient deterrents (69, 70). Political factors play a significant role, as Bangladesh’s rapid economic growth, which reached 7.2% recently, fuels urbanization and industrial activities, increasing noise pollution (71). The government often prioritizes economic development over enforcing noise regulations, especially when it involves influential sectors like construction or political activities that involve loudspeakers during election campaigns (55).

Stakeholder interests further complicate enforcement. Business owners and industries resist strict policies due to potential economic losses, while public awareness of noise pollution and its legal implications remains low (71). Even traffic police, who suffer from noise-related health issues, do not enforce regulations robustly, despite noise-induced hearing problems being common among them (72). In addition, socio-economic challenges hinder progress, as noise control measures are seen as costly, and enforcement is often viewed as secondary to economic development.

Despite all these challenges, our research suggests that effective noise pollution mitigation must not only involve better traffic management and public awareness campaigns, but also necessitate a critical evaluation and overhaul of existing noise regulation policies. Addressing enforcement gaps, increasing penalties for violations, and improving monitoring systems are essential steps to curb noise pollution in Dhakato enhance urban living standards and public health.

## 5 Conclusion

This study investigated the relationship and connection between the self-reported health status of adult populations and the observed levels of noise pollution. Exposure to noise pollution has been linked positively to health issues. The study established a comparative analysis of noise exposure according to the land use category in urban Bangladesh, where the major source of noise was generated from heavy traffic. In terms of health, residents of residential and mixed-use areas are more vulnerable to issues linked to noise pollution. Even though the study offers insightful information, more research is necessary to completely comprehend the long-term repercussions of noise pollution, particularly how it affects children, the older adult, marginalized communities, and mental health. There is room for more research to examine the financial effects of noise pollution on people, companies, and the community at large. This can entail projecting the expenses related to medical issues, missed work, and decreased property values.

While existing studies provide valuable insights into Dhaka’s noise pollution, significant gaps remain in understanding its long-term impacts. For example, while noise pollution’s effects on cardiovascular health are well-documented, research on its mental health consequences is still in its infancy (64). To fully evaluate the impact of noise exposure on psychological well-being, more extensive longitudinal studies are required, especially for susceptible populations like low-income citizens and traffic police (35). Additionally, little information exists regarding the precise impacts of noise pollution on young people, the older adult, and communities of color—a topic that has received attention in other international cities (63). Moreover, while traffic noise has been identified as a major contributor, less attention has been given to noise from other sources, such as industrial activities and public events (54). These gaps underscore the need for more targeted research, particularly on long-term health impacts and the specific vulnerabilities of marginalized communities.

To address these challenges, it is critical to implement comprehensive noise pollution control measures. Policy recommendations include strengthening enforcement of existing regulations through the creation of a dedicated noise control board and stricter penalties for violations. Campaigns for public awareness should be initiated to inform people about the dangers noise pollution poses to their health and how they may help reduce it. Furthermore, real-time data from noise monitoring infrastructure must be built so that enforcement and policy decisions may be made.

However, despite the promise of technological solutions, several challenges obstruct their successful deployment in Dhaka. High implementation costs, including installation and maintenance, present a major challenge, especially in a city with constrained financial resources. Dhaka also faces a lack of technical expertise necessary to adapt and sustain advanced noise control technologies, making local capacity-building crucial for long-term success. Gaps in regulatory enforcement further complicate matters, as the lack of strict compliance with existing noise pollution laws can undermine even the best technologies. Moreover, Dhaka’s urban infrastructure and unplanned growth exacerbate noise pollution, requiring integrated urban planning reforms alongside technological interventions to achieve meaningful change. Regulatory gaps in noise pollution enforcement in Dhaka stem from a lack of resources, poor institutional capacity, insufficient coordination among agencies, unclear policies, inadequate enforcement, corruption, lack of political commitment, and limited public awareness regarding health risks (71, 73).

Finally, social and behavioral factors pose additional barriers. Public awareness of noise pollution’s health risks remains low, and without broad understanding and cooperation, the effectiveness of technological solutions could be limited. A multi-faceted approach that includes policy enforcement, public education, infrastructure development, and investment in technical capacity is essential to overcome these challenges.

Noise pollution can be significantly decreased with the help of urban planning. This can be accomplished by promoting noise-reducing technologies in traffic and construction, implementing green buffer zones, and installing noise barriers. Technological innovations should also be encouraged through research and development initiatives.

These policy recommendations provide concrete and actionable guidelines for reducing noise pollution in Dhaka. Addressing both the financial and technical barriers is crucial, as is fostering collaboration between policymakers, urban planners, and stakeholders to ensure that noise control measures are effectively implemented. By doing so, Dhaka can move toward a quieter, healthier urban environment.

## Data Availability

The raw data supporting the conclusions of this article will be made available by the authors, without undue reservation.

## References

[1] Clark C Head J Stansfeld S. Longitudinal effects of aircraft noise exposure on Children’s health and cognition: a six-year follow-up of the UK RANCH cohort. J Environ Psychol. (2013) 35:1–9. doi: 10.1016/j.jenvp.2013.03.002

[2] Monazzam MR Nezafat A Golmahamadi R Nourollahi M. Noise characteristics of pumps at Tehran’s oil refinery and control module design. Pak J Sci Ind Res. (2009) 52:167–72.

[3] Hohmann C Grabenhenrich L de Kluizenaar Y Tischer C Heinrich J Chen C-M. Health effects of chronic noise exposure in pregnancy and childhood: a systematic review initiated by ENRIECO. Int J Hyg Environ Health. (2013) 216:217–29. doi: 10.1016/j.ijheh.2012.06.001, PMID: 22854276

[4] Monazzam M Karimi E Abbaspour M Nassiri P Taghavi L. Spatial traffic noise pollution assessment – a case study. Int J Occup Med Environ Health. (2015) 28:625–634. doi: 10.13075/ijomeh.1896.00103 26190737

[5] Chowdhury Sanjib Mahbubur Razzaque M. Helali Maksud Bodén Hans. Assessment of noise pollution in Dhaka city. Cairo, Egypt: 17th International Congress on Sound and Vibration (2010).

[6] Jahan Sayka Munni Samia Ghosh Gopal. Noise pollution at major schools, colleges and hospitals in small urban area: focusing on Jessore municipality, Bangladesh. Nat Environ Pollut Technol. (2016) 15:1089.

[7] Mamun S. Noise pollution: a bane of Bangladeshi urban life. Dhaka, Bangladesh: Dhaka Tribune. (2018).

[8] Mahbub Mehrin A. Chung Diana. Addressing environmental pollution is critical for Bangladesh’s growth and development. Text/HTML World Bank. (2024). Available at: https://www.worldbank.org/en/news/press-release/2024/03/28/addressing-environmental-pollution-is-critical-for-bangladesh-s-growth-and-development.

[9] Gössling S Humpe A Litman T Metzler D. Effects of perceived traffic risks, noise, and exhaust smells on bicyclist behaviour: an economic evaluation. Sustain For. (2019) 11:408. doi: 10.3390/su11020408

[10] Münzel T Gori T Babisch W Basner M. Cardiovascular effects of environmental noise exposure. Eur Heart J. (2014) 35:829–36. doi: 10.1093/eurheartj/ehu030, PMID: 24616334 PMC3971384

[11] Peris E. Environmental noise in Europe, 2020. LU: Publications Office (2020).

[12] Nurul A Iqbal S M AZ M AIC. Assessment of noise pollution of two vulnerable sites of Sylhet City, Bangladesh. Int J Water Resources Environ Eng. (2014) 6:112–20. doi: 10.5897/IJWREE2013.0464

[13] Rahman M Hasan L Quader M Tasnim F Rahman F Shobuj I. Perceived noise pollution and self-reported health status among adult population of Bangladesh. Int J Environ Res Public Health. (2022) 19:2394. doi: 10.3390/ijerph19042394, PMID: 35206582 PMC8872462

[14] King EA. The interaction between noise and the UN sustainable development goals - noise news international Noise News International (2022).

[15] UNEP. The World’s Cities Must Take on the Cacophony of Noise Pollution. (2022). Available at: https://www.unep.org/news-and-stories/opinion/worlds-cities-must-take-cacophony-noise-pollution.

[16] The Lancet Regional Health – Europe. Noise pollution: more attention is needed. Lancet Reg Health Eur. (2023) 24:100577. doi: 10.1016/j.lanepe.2022.100577, PMID: 36643665 PMC9832265

[17] Murphy E Faulkner JP Douglas O. Current state-of-the-art and new directions in strategic environmental noise mapping. Curr Pollut Rep. (2020) 6:54–64. doi: 10.1007/s40726-020-00141-9

[18] IANS. Noise pollution in Dhaka out of control: experts. Statesman. (2024) 2024

[19] Hoque Mir Kabir Md. Traffic induced noise level in different places at the Dhaka capital city of Bangladesh. Bangladesh J Environ Sci. (2020) 38:41–46.

[20] Ali Amina. Development and issues and megacities. DLP Forum (blog). (2023). Available at: https://www.dlpforum.org/2023/12/08/development-and-issues-of-megacities-examples-from-both-developed-and-developing-countries/.

[21] Chakma Arnab. (2023). Understanding and mitigating noise pollution in Bangladesh’s landscape.

[22] Ajibade FO Adelodun B Lasisi KH Fadare OO Ajibade TF Nwogwu NA. Chapter 25 - environmental pollution and their socioeconomic impacts In: Kumar A Singh VK Singh P Mishra VK, editors. Microbe mediated remediation of environmental contaminants: Woodhead Publishing Series in Food Science, Technology and Nutrition. Woodhead Publishing (2021). 321–54.

[23] Slabbekoorn H. Noise pollution. Curr Biol. (2019) 29:R957–60. doi: 10.1016/j.cub.2019.07.018 31593676

[24] Thompson R Smith RB Karim YB Shen C Drummond K Teng C. Noise pollution and human cognition: an updated systematic review and *Meta*-analysis of recent evidence. Environ Int. (2022) 158:106905. doi: 10.1016/j.envint.2021.106905, PMID: 34649047

[25] Clark C Paunovic K. WHO environmental noise guidelines for the European region: a systematic review on environmental noise and quality of life, wellbeing and mental health. Int J Environ Res Public Health. (2018) 15:2400. doi: 10.3390/ijerph15112400, PMID: 30380665 PMC6266190

[26] Eriksson Charlotta Pershagen Göran. Biological mechanisms related to cardiovascular and metabolic effects by environmental noise. Europe: World Health Organization (2018).

[27] Hänninen O Knol AB Jantunen M Lim T-A Conrad A Rappolder M. Environmental burden of disease in Europe: assessing nine risk factors in six countries. Environ Health Perspect. (2014) 122:439–46. doi: 10.1289/ehp.1206154, PMID: 24584099 PMC4014759

[28] Marquart H Ueberham M Schlink U. Extending the dimensions of personal exposure assessment: a methodological discussion on perceived and measured noise and air pollution in traffic. J Transp Geogr. (2021) 93:103085. doi: 10.1016/j.jtrangeo.2021.103085

[29] Idler EL Benyamini Y. Self-rated health and mortality: a review of twenty-seven community studies. J Health Soc Behav. (1997) 38:21–37. doi: 10.2307/2955359, PMID: 9097506

[30] Ou JY Peters JL Levy JI Bongiovanni R Rossini A Scammell MK. Self-rated health and its association with perceived environmental hazards, the social environment, and cultural stressors in an environmental justice population. BMC Public Health. (2018) 18:970. doi: 10.1186/s12889-018-5797-7, PMID: 30075713 PMC6090753

[31] Caramenti M Castiglioni I. Determinants of self-perceived health: the importance of physical well-being but also of mental health and cognitive functioning. Behav. Sci. (2022) 12:498. doi: 10.3390/bs12120498, PMID: 36546981 PMC9774654

[32] Riedel N van Kamp I Dreger S Gabriele Bolte TC Andringa SP Schreckenberg D. Considering ‘non-acoustic factors’ as social and environmental determinants of health equity and environmental justice. Reflections on research and fields of Action towards a vision for environmental noise policies. Trans Res Interdisciplin Perspect. (2021) 11:100445. doi: 10.1016/j.trip.2021.100445

[33] Szombathely V Malte MA Augustin J Bechtel B Dwinger I Gaffron P. Relation between observed and perceived traffic noise and socio-economic status in urban blocks of different characteristics. Urban Sci. (2018) 2:20. doi: 10.3390/urbansci2010020

[34] Herrera C Cabrera-Barona P. Impact of perceptions of air pollution and noise on subjective well-being and health. Earth. (2022) 3:825–38. doi: 10.3390/earth3030047

[35] Newbury JB Heron J Kirkbride JB Fisher HL Bakolis I Boyd A. Air and noise pollution exposure in early life and mental health from adolescence to young adulthood. JAMA Netw Open. (2024) 7:e2412169. doi: 10.1001/jamanetworkopen.2024.12169, PMID: 38805229 PMC11134215

[36] Krejcie RV Morgan DW. Determining sample size for research activities. Educ Psychol Meas. (1970) 30:607–10. doi: 10.1177/001316447003000308

[37] Manualslib. UNI-T UT351 OPERATING MANUAL pdf download. ManualsLib Accessed. (2024). Available at: https://www.manualslib.com/manual/538680/Uni-T-Ut351.html.

[38] Bonet-Solà D Bergadà P Dorca E Martínez-Suquía C Alsina-Pagès RM. Sons al Balcó: a comparative analysis of WASN-based LAeq measured values with perceptual questionnaires in Barcelona during the COVID-19 lockdown. Sensors. (2024) 24:1650. doi: 10.3390/s24051650, PMID: 38475185 PMC10935095

[39] Chen X Liu M Zuo L Xiaoyi W Chen M Li X. Environmental noise exposure and health outcomes: an umbrella review of systematic reviews and Meta-analysis. Eur J Pub Health. (2023) 33:725–31. doi: 10.1093/eurpub/ckad044, PMID: 37030015 PMC11314258

[40] Kumari S Sharma A Ghosh AK. A comprehensive review of noise pollution monitoring studies at bus transit terminals. Noise Mapping. (2024) 11:20220180. doi: 10.1515/noise-2022-0180

[41] Leaffer DJ Suh H Durant JL Tracey B Roof C Gute DM. Long-term measurement study of urban environmental low frequency noise. J Expo Sci Environ Epidemiol. (2023):1–13. doi: 10.1038/s41370-023-00599-x 37696975

[42] Nazneen S Raza A Khan S. Assessment of noise pollution and associated subjective health complaints and psychological symptoms: analysis through structure equation model. Environ Sci Pollut Res. (2020) 27:21570–80. doi: 10.1007/s11356-020-08655-x, PMID: 32279247

[43] SAVEnviron. Estimation of equivalent noise level - YouTube. (2021). Available at: https://www.youtube.com/watch?v=K-tzydTuGeg.

[44] Majidi F Khosravi Y Abedi K. Determination of the equivalent continuous sound level (Leq) in industrial indoor space using GIS-based noise mapping. J Hum Environ Health Promotion. (2019) 5:50–5. doi: 10.29252/jhehp.5.2.1

[45] Waller G. Self-rated health: from epidemiology to patient encounter. Umeå: Department of Public Health and Clinical Medicine, Umeå University (2015).

[46] Fayers PM Sprangers MAG. Understanding self-rated health. Lancet. (2002) 359:187–8. doi: 10.1016/S0140-6736(02)07466-4 11812551

[47] Strawbridge WJ Wallhagen MI. Self-rated health and mortality over three decades: results from a time-dependent covariate analysis. Res Aging. (1999) 21:402–16. doi: 10.1177/0164027599213003

[48] Galenkamp Henrike Braam Arjan W. Huisman Martijn Deeg Dorly J. H. “Self-rated health: when and how to use it in studies among older people?” In International handbook of health expectancies, edited by Jagger Carol Crimmins Eileen M. Saito Yasuhiko Yokota Renata Tiene De Carvalho Oyen Herman Van Robine Jean-Marie, 173–181. Cham: Springer International Publishing. (2020).

[49] ECR. (1997). Environment Conservation Rules, 1997. UNEP Law and Environment Assistance Platform.

[50] NPCR. Noise pollution control rules. (2006). Available at: https://file-mymensingh.portal.gov.bd/uploads/0c00a35e-4eb1-444d-b753-9394a7707ac8//632/7e7/316/6327e7316b081676413857.pdf.

[51] RTA. “সড়ক পরিবহণ আইন, ২০১৮. (2018). Available at: http://bdlaws.minlaw.gov.bd/act-1262.html.

[52] George D Mallery P. IBM SPSS statistics 26 step by step: a simple guide and reference. 16th ed. New York: Routledge (2019).

[53] Python. Welcome to Python.Org. Python.Org. (2024). Available at: https://www.python.org/.

[54] Razzaque M Mahbubur Chowdhury Sanjib Chandra Helali Maksud Bodén Hans. On the impacts of noise pollution in Dhaka. Cairo, Egypt: 17th International Congress on Sound and Vibration 2010, ICSV 2010 (2010).

[55] Dey Amit Ranjan. Noise pollution in Dhaka: current situation and suggestions for action. (2016). Available at: https://ben-global.net/wp-content/uploads/2016/08/Noise-Pollution-In-Dhaka-Current-Situation-And-Suggestions-For-Action-2002.pdf.

[56] Prothom Alo. Dhaka tops the noise pollution index too. Prothom Alo. (2022). Available at: https://en.prothomalo.com/environment/dhaka-tops-the-noise-pollution-index-too.

[57] Riyad R Amin A Mazumder M. A study of noise pollution by traffic during peak and off peak hour in Dhaka City (2020) 2:43–53.

[58] Numbeo. Pollution comparison between Mumbai, India and Dhaka, Bangladesh. (2024). Available at: https://www.numbeo.com/pollution/compare_cities.jsp?country1=India&city1=Mumbai&country2=Bangladesh&city2=Dhaka.

[59] Jahan DS. Noise pollution and its probable impacts on public health in Dhaka City. (2022). Available at: https://pressinform.portal.gov.bd/sites/default/files/files/pressinform.portal.gov.bd/page/50aa82ee_7a92_4aa7_817b_bb323c098833/2022-12-07-06-14-a4421b30fd079a95cdb934e3193f5b70.pdf.

[60] Hahad O Kuntic M Al-Kindi S Kuntic I Gilan D Petrowski K. Noise and mental health: evidence, mechanisms, and consequences. J Expo Sci Environ Epidemiol. (2024):1–8. doi: 10.1038/s41370-024-00642-5 38279032 PMC11876073

[61] Nega TH Chihara L Smith K Jayaraman M. Traffic noise and inequality in the Twin Cities, Minnesota. Hum Ecol Risk Assess Int J. (2013) 19:601–19. doi: 10.1080/10807039.2012.691409

[62] Warner SC Sagovac S Godwin C Xia T Batterman S. Community’s perception on ambient air and noise pollution: a qualitative study in Southwest Detroit. Environ Justice. (2023) 16:286–96. doi: 10.1089/env.2021.0085, PMID: 37614719 PMC10443084

[63] Nelson-Olivieri JR Layden TJ Antunez E Khalighifar A Lasky M Laverty TM. Inequalities in noise will affect urban wildlife. Nat Ecol Evol. (2024) 8:163–74. doi: 10.1038/s41559-023-02257-9, PMID: 37985897

[64] Gong X Fenech B Blackmore C Chen Y Rodgers G Gulliver J. Association between noise annoyance and mental health outcomes: a systematic review and Meta-analysis. Int J Environ Res Public Health. (2022) 19:2696. doi: 10.3390/ijerph19052696, PMID: 35270388 PMC8910193

[65] Hajat A Hsia C O’Neill MS. Socioeconomic disparities and air pollution exposure: a global review. Curr Environ Health Rep. (2015) 2:440–50. doi: 10.1007/s40572-015-0069-5, PMID: 26381684 PMC4626327

[66] Evans BE Tuvblad C Larsson H. Urban living and mental health. Nat Med. (2023) 29:1322–3. doi: 10.1038/s41591-023-02348-x 37322118

[67] BD Welfare Society. Noise pollution in Bangladesh: alarming health impacts and solutions. (2024). Available at: https://www.bdwelfaresociety.org/noise-pollution-in-bangladesh/.

[68] Roy Shilajit Kumar. Urban soundscapes: understanding noise pollution in Bangladesh. LawyersclubbangladeshCom (blog). (2023). Available at: https://lawyersclubbangladesh.com/en/2023/10/12/urban-soundscapes-understanding-noise-pollution-in-bangladesh/.

[69] Faroque S South N. Law-enforcement challenges, responses and collaborations concerning environmental crimes and harms in Bangladesh. Int J Offender Ther Comp Criminol. (2022) 66:389–406. doi: 10.1177/0306624X20969938, PMID: 33153345 PMC8807541

[70] The Daily Star. Looking into the noise pollution (control) rules. The Daily Star. (2019). Available at: https://www.thedailystar.net/law-our-rights/news/looking-the-noise-pollution-control-rules-1847602.

[71] Karim ME Taher MA Karim MA. Noise pollution in Dhaka and the constitutional right to life. SSRN Scholarly Paper. Rochester, NY. (2021). Available at: https://papers.ssrn.com/abstract=3875921.

[72] Dhaka Tribune. Noise pollution: will Dhaka ever be quiet? Dhaka Tribune. (2021). Available at: https://www.dhakatribune.com/bangladesh/dhaka/236683/noise-pollution-will-dhaka-ever-be-quiet.

[73] Ahmed T Rahman T. Non-auditory health Hazard vulnerability to noise pollution: assessing public awareness gap. Am J Eng Res. (2015) 4:143–7.

